# Editorial: Sustainable development in the agro-food and nutrition sector

**DOI:** 10.3389/fnut.2024.1419937

**Published:** 2024-05-29

**Authors:** Monika Thakur, Claudio Ferrante, Ana Sanches Silva

**Affiliations:** ^1^Amity Institute of Food Technology, Amity University Uttar Pradesh, Noida, India; ^2^Department of Pharmacy, G. d'Annunzio University of Chieti-Pescara, Chieti, Italy; ^3^Faculty of Pharmacy, University of Coimbra, Coimbra, Portugal; ^4^Centre for Study in Animal Science (CECA), ICETA, University of Porto, Porto, Portugal; ^5^Associate Laboratory for Animal and Veterinary Sciences (AL4AnimalS), Lisbon, Portugal

**Keywords:** sustainable diets, sustainable food production, food policies, food marketing, SDGs, Food system, environmental sustainability

As the world population continues to grow, much more effort and innovation will be urgently needed in order to sustainably increase agricultural production, improve the global supply chain, decrease food losses and waste, and ensure that all who are suffering from hunger and malnutrition have access to nutritious food. Many in the international community believe that it is possible to eradicate hunger within the next generation, and are working together to achieve this goal. The imperative of sustainable development within the agro-food and nutrition sector are essential for an equitable and healthy society. The convergence of global pandemics, exacerbated inequality, climate instability, and continuous mounting food demands presents an acute menace to global food security. The world is facing an impending food crisis, with nearly 30% of the global population lacking regular access to sufficient food (1).

Modern innovative technologies offer unique opportunities to revolutionize food production, enhance quality, optimize supply chain management, and invigorate commerce, thereby addressing the myriad technological and economic challenges afflicting the agro-food industry. The transformative strides catalyzed by sustainable development initiatives within food and agriculture systems are pivotal in charting our course toward the attainment of the United Nations Sustainable Development Goals (SDGs) by 2030. The adoption of sustainable practices holds the promise of significantly amplifying agricultural yields with diminished human intervention, fostering a comprehensive sustainability ethos spanning economic, social, and environmental realms (2, 3).

Beyond adequate calories intake, proper nutrition has other dimensions that deserve attention, including micronutrient availability and healthy diets. Inadequate micronutrient intake of mothers and infants can have long-term developmental impacts. Unhealthy diets and lifestyles are closely linked to the growing incidence of non-communicable diseases in developed and developing countries. To cultivate sustainability, agriculture must deftly navigate the exigencies of present and future demands, all while ensuring profitability, environmental integrity, and socioeconomic parity. Sustainable Food and Agriculture (SFA) play a pivotal role in advancing all four pillars of food security: availability, accessibility, utilization, and stability—while simultaneously engendering a holistic sustainability paradigm that encompasses environmental stewardship, social equity, and economic viability. The resounding endorsement of SFA by the Food and Agriculture Organization (FAO) reverberates globally, poised to propel nations toward the formidable goal of Zero Hunger and the realization of the SDGs (4).

The agro-food and nutrition sectors confront unparalleled challenges stemming from escalating global population demands, burgeoning hunger and malnutrition rates, the deleterious impacts of climate volatility, unabated natural resource exploitation, biodiversity erosion, and the scourge of food loss and waste. These formidable hurdles imperil our collective capacity to meet extant and future food needs, resulting in inadequate access to nutritious food for many individuals.

Galvanized by the imperative of enhancing the global supply chain, curbing food loss and waste, and fortifying agricultural production in a sustainable fashion, concerted efforts are underway to surmount the exigencies posed by a burgeoning global population. Collaborative initiatives resonate across the globe, propelled by an unwavering belief in the achievability of eradicating hunger and malnutrition. In addition to calorie sufficiency, maintaining a balanced diet and ensuring micronutrient availability are crucial aspects of good nutrition that should not be overlooked. Inadequate consumption of essential micronutrients among mothers and infants carries profound developmental repercussions, while the escalating prevalence of non-communicable diseases in both affluent and developing nations underscores the intimate nexus between diet, lifestyle, and health outcomes across diverse socio-economic spectra.

Global Agricultural Systems must transition toward increased productivity and reduced wastefulness. Sustainable agricultural paradigms and food systems necessitate a comprehensive, integrated approach that holistically addresses the manifold facets of production and consumption. The international community is exhorted to bolster investments in research, innovation, and technology demonstration to enhance the sustainability quotient of global food systems amidst the specter of climate change-induced perturbations.

Efforts to ensure food security and optimal nutrition for all hinge on strengthening the resilience of regional food systems and preventing widespread shortages in the future. With agro-food systems worldwide grappling with the challenge of provisioning for an expanding populace, these emergent innovations offer a beacon of hope for a healthier and more equitable nutritional future.

The study focus is on Sustainable development in agri & food sector (Figure 1). Recent research endeavors have further elucidated these themes:

- Thakur et al. delved into marketing performance and the determinants influencing farmers' selection of agricultural output marketing channels, with a specific focus on garden pea production in India. This study aimed to examine the marketing performance and factors influencing farmers choice for agricultural output marketing channels in garden pea in the Indian state of Himachal Pradesh.
- Avnee et al. expounded on biofortification as a strategic intervention to combat micronutrient deficiencies, particularly pervasive in developing nations. The research highlights the deficiencies which have been particularly common in developing countries, where a lack of access to a varied and nutritious diet makes it difficult for people to get the micronutrients they need.
- Filipa-Silva et al. conducted a meticulous evaluation of alternative marine lipid sources for substituting fish oil in sea bass, discerning their impact on product quality during cold storage. Their study evaluated the replacement of fish oil (sardine oil) by different combinations of alternative marine lipid ingredients as sources of eicosapentaenoic acid (EPA) and docosahexaenoic acid (DHA) in European sea bass (*Dicentrarchus labrax*) throughout 14 days of ice storage.
- Nguyen et al. scrutinized the environmental ramifications and nutrient adequacy of dietary patterns in Vietnam. Despite that diet quality was slightly better in all three patterns compared to the average diet of the total population, environmental impact was also higher. Therefore, future research is needed to develop a more optimal diet that considers both diet quality and environmental impact to explore the trade-offs between diet quality and environmental impact.
- Valdiviezo-Marcelo et al. probed the technological potential of native lactic acid bacteria in artisanal cheese production in Peru, unraveling novel avenues for augmenting sensory attributes. Their research focuses on interesting scenario for the development of new research in cheese formulation using combinations of isolated bacterial strains together with commercial starter cultures, with increased sensory accept abilities.
- Cao and Li illuminated the nexus between healthy dietary practices and household food waste, underlining the instrumental role of health awareness in ameliorating food wastage. The results showed that greater awareness of what constitutes a healthy diet can significantly reduce household food waste.
- Raghavendra et al. investigated the determinants of integrated farming systems adoption and their transformative impact on livelihoods and dietary diversity. The findings of the study indicate that age, education, livestock holding, access to credit, and plantation area have a positive and significant effect on participation by farmers in the program.

**Figure 1:**
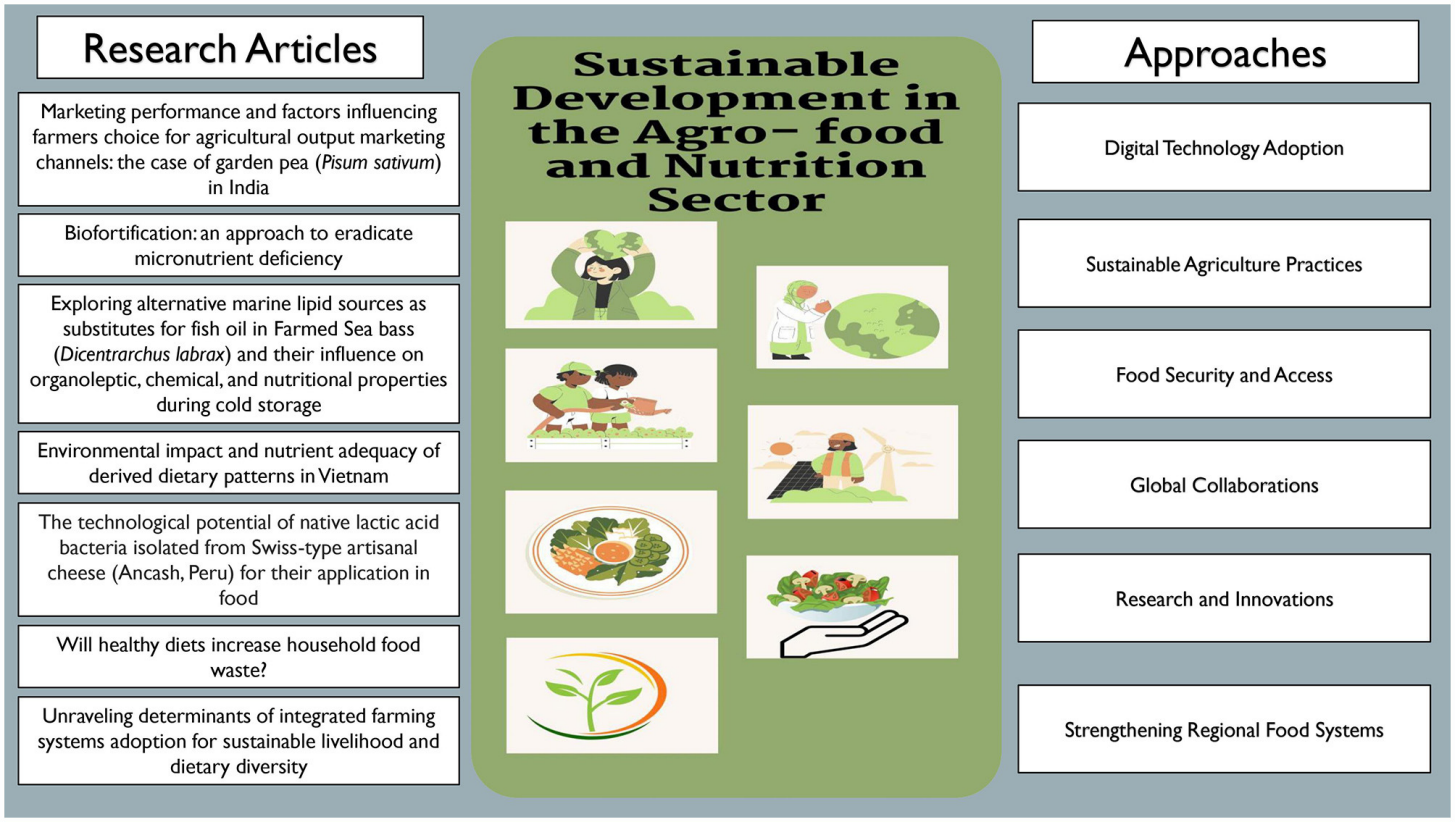
Sustainable development in agro-food and nutrition sector with different parameters for opportunities and approaches.

The ethos of sustainable nutrition epitomizes an optimal, health-enhancing dietary regimen that is culturally resonant, readily accessible, and ecologically benign, with an eye toward mitigating ecological footprints for both the present and future generations.

The Research Topic entitled “*Sustainable development in the agro- food and nutrition sector*” explores the intersection of sustainable development with agriculture, food production, and nutrition, highlighting the importance of balancing economic viability with environmental stewardship and presents a comprehensive Research Topic of original research, reviews, and market reviews that encompass various facets of recent advancements in the field. The readers will recognize the diverse nature of the challenges and opportunities to explore the new avenues of research, thereby contributing to the development of sustainable system in *Agro- food and Nutrition Sector*. Such efforts align with the SDG's play a pivotal and crucial role in enhancing production, nutrition, environmental sustainability, and overall quality of life.

It showcases innovative technologies, policy interventions, and research initiatives driving progress toward sustainable food systems that can meet the needs of present and future generations while safeguarding planetary health. These endeavors are instrumental in advancing production, nutrition, environmental sustainability, and overall quality of life in alignment with the SDGs. Therefore, gaining insight into the challenges and opportunities within food science and technology is crucial, as it directly influences our capacity to attain SDGs, especially those pertaining to food security, health, and sustainability. By leveraging innovation and research in this domain, we can strive toward establishing more efficient, sustainable, and equitable food systems, thereby propelling progress toward the overarching global objectives of the SDGs by 2030.

In conclusion, the integration of sustainable development principles within the agro-food and nutrition sector is paramount for fostering global resilience, equity, and health. As we confront multifaceted challenges such as climate change, food insecurity, and malnutrition, embracing sustainable practices and technological innovations offers a transformative pathway toward a more equitable and nourished future for humanity. Collaboration, research, and policy interventions are essential in realizing the vision of sustainable food systems that can meet the needs of current and future generations without compromising the integrity of our planet.

## Author contributions

MT: Conceptualization, Resources, Supervision, Validation, Writing – original draft, Writing – review & editing. CF: Conceptualization, Investigation, Writing – review & editing. AS: Conceptualization, Investigation, Methodology, Writing – review & editing.
